# Perspective on Cerebral Autoregulation Monitoring in Neonatal Cardiac Surgery Requiring Cardiopulmonary Bypass

**DOI:** 10.3389/fneur.2021.740185

**Published:** 2021-10-05

**Authors:** Jared M. Spilka, Conor P. O'Halloran, Bradley S. Marino, Kenneth M. Brady

**Affiliations:** ^1^Division of Cardiac Anesthesia, Department of Anesthesiology, Ann and Robert H. Lurie Children's Hospital of Chicago, Chicago, IL, United States; ^2^Division of Cardiology, Department of Pediatrics, Ann and Robert H. Lurie Children's Hospital of Chicago, Chicago, IL, United States; ^3^Department of Pediatric Cardiology, Cleveland Clinic, Cleveland, OH, United States

**Keywords:** cerebral autogregulation, congenital heart disease, neonate, autoregulation monitoring, pediatrics, neurological injury, cardiopulmonary bypass

## Abstract

The autoregulation of cerebral blood flow protects against brain injury from transient fluctuations in arterial blood pressure. Impaired autoregulation may contribute to hypoperfusion injury in neonates and infants. Monitoring cerebral autoregulation in neonatal cardiac surgery as a guide for arterial blood pressure management may reduce neurodevelopmental morbidity. Cerebral autoregulation monitoring has been validated in animal models and in an adult trial autoregulation monitoring during bypass improved postoperative delirium scores. The nuances of pediatric cardiac disease and congenital heart surgery make simply applying adult trial findings to this unique population inappropriate. Therefore, dedicated pediatric clinical trials of cerebral autoregulation monitoring are indicated.

## The Burden of Neurologic Injury After Neonatal Heart Surgery

Neurological injury occurs in roughly half of newborns undergoing cardiac surgery using cardiopulmonary bypass (CPB) (1). MRI studies reveal that stroke occurs in about 10% of this population (2) secondary to both ischemic and thromboembolic events. However, white matter injury (WMI) occurs at a much higher rate, affecting over 50% of neonates undergoing cardiac surgery with CPB +/- deep hypothermic circulatory arrest (DHCA) vs. only 4% in infants who require similar surgical procedures (3). The potential causes of inadequate perfusion instigating WMI are myriad. Neonates with critical congenital heart disease have oxygen delivery vulnerabilities *in utero*, preoperatively, intraoperatively, and postoperatively. Highly sensitive oligodendrocytes occupy a watershed region in the neonatal brain, and this is thought to contribute to the high incidence of white matter injury reminiscent of preterm periventricular leukomalacia (4). The duration of CPB, use and duration of DHCA, low arterial blood pressure (ABP), and hypoxemia in the first 48 post-operative hours are all associated with WMI (3, 5). The most complex congenital cardiac lesions require repair by a series of staged surgeries, potentially compounding injury over time through multiple episodes of CPB. The magnitude of disability associated with WMI is not yet established and cannot be studied without following cohorts to teenage and adult years. A recent, large meta-analysis of neurodevelopmental outcome after neonatal cardiac surgery showed a disappointing magnitude of impairment in both the psychomotor and cognitive indices of the Bayley Scale of Infant Development (78 and 88% of normative means on average). (6) Improved understanding of neonatal brain perfusion and interventions to reduce neurological injury have high potential impact for neonates who require heart surgery and CPB.

### Watershed Injuries in the White Matter Suggest Vulnerability to Inadequate Cerebral Blood Flow

Neonates, especially preterm neonates, lack the robust, redundant cerebral vasculature that is present in infants and adults. Poor vascular redundancy in the deep white matter may enhance the risk of watershed injury during cerebral dysautoregulation. Fragile vascular overgrowth in the germinal matrix is a risk factor for hemorrhage for the preterm neonate, but germinal matrix hemorrhage is a less common injury after CPB than watershed WMI. (7) Congenital heart disease is associated with increased risk of abnormal brain development, independent of the finding of brain injury during surgical or procedural interventions. Neonates with congenital heart disease have small, relatively immature brains, perturbations in brain structural integrity and metabolism, regional growth differences, and an overall decrease in cerebral blood flow (8–12). The immaturity of the neurovascular system in neonates with congenital heart disease may contribute to both dysautoregulation of cerebral blood flow, and vulnerability to injury from dysautoregulation. Prevention of the dysautoregulated state by maintaining ABP within the limits of autoregulation is a modifiable care practice that may prevent or mitigate WMI in this population.

### Hypotension, and Optimal ABP for Neonates With Heart Disease Are Unknown

The goal of autoregulation monitoring in neonates with congenital heart disease is to delineate the lower limit of autoregulation (LLA), providing a target for ABP management. (7) Maintaining ABP above LLA is hoped to protect watershed regions of the neonatal brain from fluctuations of cerebral blood flow that occur in the dysautoregulated state. (13) The limits of autoregulation for neonates, and the inter-subject variability of these limits are not known. Criteria for hypotension in neonates are poorly defined and best treatments of “hypotension” (fluid, vasopressors, ionotropes) are debated (14) Current ABP management is based solely on experiential knowledge rather than individualized physiological goals (7). Further, there is a dichotomy of physiological goals which occurs in the setting of neonatal cardiac pathology, which benefits from low ABP and maintaining cerebral autoregulation, which may require higher ABP. When the neonatal heart is weakened by surgery and critical illness, lowered systemic vascular resistance (SVR) improves stroke volume and cardiac output. There is no effective measure of SVR, stroke volume or cardiac output for neonates, so lowered ABP is used as a primary surrogate. Strict SVR control has improved outcomes for neonates with the most critical heart lesions (13, 15, 16). However, the brain is dependent on ABP, not cardiac output for perfusion, so this highly effective ABP lowering strategy may improve survival at the expense of an increase in brain injury.

In principle the maintenance of ABP above LLA during neonatal cardiac surgery may prevent neurological injury and improve neurodevelopmental outcomes. This remains as an only partially tested hypothesis which requires further study (13). Cerebral autoregulation monitoring during CPB in adults has shown improvement in neurological outcomes (17, 18). The uniqueness of neonatal physiology and the requirement for strict SVR control when heart disease is present are factors that elevate the importance of studying the limits of autoregulation in this population.

### Technical Considerations for Autoregulation Monitoring in the Cardiac OR

The concept of autoregulation pre-dates the technical ability to measure autoregulation in real time. Conceptually, a real time autoregulation monitor requires a measure of arterial blood pressure (ABP) or cerebral perfusion pressure (CPP), a measure of cerebral blood flow (CBF) or a proxy, and a mathematical approach to determine if cerebral blood flow is passive to blood pressure (19).

The first modern attempts to monitor autoregulation in real time are typified by Czosnyka et al. in 2 seminal papers (20, 21). These methods utilized transcranial Doppler (the mean velocity index or Mx) or intracranial pressure (the pressure reactivity index or PRx). Czosnyka et al. explained that the positive correlation between middle cerebral artery flow velocity and CPP (i.e., positive Mx) was evidence of pressure passive cerebral blood flow (i.e., decreased ABP leads to decreased CBF) which defines disturbed autoregulation. Utilizing the same cohort and study design, ICP was used as a surrogate for cerebral blood volume to quantify vasoreactivity as passivity of blood volume to ABP (20). During normal cerebral vasoreactivity, a negative relationship between ABP and ICP should be observed (lower ABP leads to vasodilation, leads to higher blood volume, leads to higher ICP). Conversely, dysfunctional vasoreactivity renders a positive relationship between ABP and ICP (lower ABP, vascular tone does not change, blood volume decreases due to lower distending pressure, ICP decreases). These methods are ideal for patients with head trauma, but trans-cranial Doppler insonation under a surgical drape is difficult during neonatal heart surgery, and invasive intracranial pressure monitoring is not possible.

### Two Requirements for the Cardiac OR: Hands-Free and Non-Invasive

The feasibility of less invasive monitors of cerebral pressure autoregulation using reflectance near infrared spectroscopy (NIRS) was established in a series of experiments in neonatal piglets (22, 23). In these experiments a “gold standard” autoregulation curve was established using invasive laser Doppler flow probes. Relative total hemoglobin (rTHb) was measured using NIRS as the inverse of the recovery of light isosbestic to the hemoglobin species. Whereas PRx correlates ABP with ICP, Hemoglobin Volume Index (HVx) correlated ABP with NIRS measure of total hemoglobin, which is a proxy for cerebral vascular tone and ICP. rTHb measured in this way served as a proxy for cerebral vascular tone to render a NIRS-based analog of the PRx, the Hemoglobin-Volume Index, or HVx. Additionally, standard, commercially available measures of cerebral tissue oxygenation were used as a proxy for CBF to render a NIRS-based analog of the Mx, the Cerebral Oximetry Index, or COx. Both the HVx and the COx were compared against the gold standard LLA. Receiver operator curves (ROC) describing the ability of HVx and COx to distinguish autoregulation from dysregulation were created.

Two relevant observations came from these experiments. First, rTHb showed high coherence (relatedness in the frequency domain) with ICP at the slow wave frequency. This observation inadvertently validated the assumption that ICP slow waves are the result of fluctuating CBV, which is an assumption inherent to the PRx. Second, non-invasive measures of autoregulation (COx) and cerebral vasoreactivity (HVx) effectively differentiate periods of cerebral autoregulation from dysregulation with similar performance to invasive measures (i.e., PRx). For example, the area under the curve (AUC) of the ROC for HVx was 0.85, compared to an AUC of 0.88 for the invasive measure PRx. HVx had a sensitivity of 77% and specificity of 84%. COx had an AUC of 0.89, sensitivity of 83%, and specificity of 75%.

### Why Frequency Matters?

In their pioneering work, Czosnyka et al. discussed the importance of examining the correct frequency of waveform when monitoring cerebral autoregulation. ABP fluctuates over each second due to cardiac systole/diastole, every few seconds due to respirations, and over minutes to hours due to changes in clinical status. In addition, ABP fluctuates every 30 seconds to few minutes in a phenomenon termed slow waves. These waves were first described in the ICP pressure tracing and were termed B waves (early observations of cerebral autoregulation were seen by visual comparison of ABP and ICP tracings at the B wave frequency) (24). Slow waves are ideal for measuring autoregulation because they are naturally occurring blood pressure fluctuations that occur at a frequency that engages the autoregulation mechanism. By contrast, the ABP pulse due to cardiac systole/diastole occurs too rapidly and is not buffered by cerebral autoregulation. The respiratory cycle in ABP is also too rapid for reliable engagement of the autoregulation mechanism, especially at rapid respiratory rates seen in neonates. Therefore, slow wave oscillations in ABP have been used to measure autoregulation and vasoreactivity in most attempts to quantify autoregulation in real time. The use of slow waves dictates the refresh rate of autoregulation monitoring, and is the rate-limiter for time to delineate the lower limit of autoregulation. Current methods are estimated to require 15 to 30 min of monitoring to delineate the lower limit of autoregulation in the neonatal cardiac operating room.

### Too Many Options: How Do We Validate Them?

The number of proposed measures of autoregulation and vasoreactivity has increased in the last decade, with varying levels of evidence to support their utilization. All metrics share 3 fundamental components (25):

1) **Changes in ABP must occur and be measured**. Measurement of ABP is straight forward using intra-arterial catheters which are standard practice in intensive care units. Most attempts to measure autoregulation have focused on the slow wave oscillations in ABP described above.
2) **Changes in CBF or CBV must be measured**. A number of different non-invasive monitors of CBF or CBV have been proposed, most of which rely on measuring blood flow by doppler effect, or oximetry and blood volume based on the light absorption of hemoglobin.
3) **The relationship between ABP and CBF/CBV must be quantified to identify periods when CBF/CBV are passive to changes in ABP**. Of the three components this is the least intuitive to most clinicians. Conceptually all measures of relatedness can be divided into time-domain and frequency-domain measures. Time domain measures of relatedness determine if and to what extent two variables “move together” over time. The Pearson correlation coefficient is the most commonly used time-domain metric. Alternatively, complex biologic signals such as ABP or cerebral tissue oxygenation can be broken down into combinations of waves of different frequencies and magnitudes. In an oversimplification, frequency domain measures determine if and to what degree the component waves that make up more complex signals are shared between different signals (i.e., between ABP and cerebral oxygenation).

Although the NIRS based measures utilizing correlation or phase shift have performed well in animal validation, there are several other potential measures of CBF/CBV and mathematical approaches to assessing passivity to blood pressure. This leads to an essentially endless list of possible combinations. With so many measures of autoregulation (and more likely to be developed), it is important to consider the evidence burden needed to compare and optimize these potential tools. The following three requirements have been proposed (26):

1) Validate the candidate index against an accepted gold standard measure of autoregulation (in pediatric cardiac surgery population, this would be an autoregulation curve calculated from laser doppler probe and arterial blood pressure). Given that gold standard measures of cerebral blood flow are invasive, this criterium is likely to require animal study.
2) Show strong relatedness of a candidate index to outcome reproducibly in prospective studies of humans.
3) Demonstrate feasibility of using actionable data from a candidate index, such that clinicians can optimize clinical care and minimize morbidity.

### Surprisingly Poor Performances in Some Published Metrics When Studied Methodically

Utilizing the piglet experimental construct described above, a large number of candidate indices were compared with each other and to the gold standard LLA (19). The available measures included tissue oxygenation measured by NIRS (rSO2), total hemoglobin measured by NIRS (THb, measured using the isosbestic wavelength method), laser doppler flow (LDF), ICP, and mean arterial blood pressure (MAP). Four mathematical approaches (correlation, phase shift, gain of transfer and coherence) were applied to 6 variable pairs to create 24 indices of autoregulation. Receiver operator curves were created for each index (using the gold standard LLA) and area under the curves estimated to allow comparisons. These receiver operator curves determine the ability of each index to correctly discriminate between times within the ABP bounds of autoregulation (as determined by laser doppler probe-ABP relationship) and times outside the ABP bounds of autoregulation. Eight indices appeared to outperform the other metrics. This tedious, but important analysis showed that correlation and phase- based indices clearly outperformed those based on either gain of transfer or coherence. NIRS-derived measures of cerebral oximetry and blood volume (ie rTHb) performed as well as invasive ICP and out-performed direct flow measurements with laser-Doppler. Any new method to measure autoregulation, either by novel interrogation of the brain vasculature or novel mathematic assessment of passivity to ABP needs to undergo a similar evaluation as many published methods failed to delineate LLA when critically examined this way.

### NIRS Can Measure Autoregulation. Is It Feasible in the Neonatal Cardiac OR?

HVx and COx are candidate indices that meet the requirements of being hands-free and non-invasive. Both performed well in the animal validation study. However, neither has been studied adequately in the clinical environment to demonstrate feasibility or efficacy to prevent neurologic injury in the neonatal cardiac population. As a start to this effort, the feasibility NIRS-based methods to measure autoregulation during cardiac surgery in children was demonstrated in a prospective observational pilot study of 54 children (13). The LLA was estimated using a COx threshold of 0.4 (extrapolated from the piglet data) and was able to be determined for 77% of the subjects during surgery. The average LLA was 42 mmHg but ranged from 20 to 55 mmHg (including non-neonates). This study demonstrated that LLA could be estimated in the clinical environment, but did not demonstrate relatedness to outcome, and did not demonstrate true feasibility: ie, if the LLA is known, is it actionable? How long did it take to delineate LLA? Can the burden of hypotension be reduced by knowing the LLA? One true demonstration of autoregulation monitoring feasibility using the mean velocity index (Mx) has been done in an adult population. Hogue et al. significantly reduced, by nearly half, the magnitude and duration of ABP below LLA when LLA was revealed to the care team with real time monitoring (18). The area-under-curve variable used in that study quantifies the burden of hypotension below LLA and is ideal for studying feasibility of autoregulation monitoring for the neonatal cardiac surgical population. If LLA can be known in a timely manner, and if the care team has options to raise ABP above LLA, then the hypotension exposure will be lower.

### Safety Concerns in the Neonatal Population

As described above, function of the neonatal heart is negatively impacted by afterload in the perioperative window. Raising ABP to exceed LLA is an expected intervention from showing LLA to care providers with autoregulation monitoring. Options include increasing pump flow rates, contractility, preload, and systemic vascular resistance. Each of these has the potential for harm. Does optimizing perfusion of the brain also optimize cardiac performance and perfusion of the visceral organs? It seems unlikely given that the brain is dependent on ABP for perfusion while the viscera are dependent on cardiac output (27, 28). If ABP maintenance comes at the expense of cardiac output, then higher rates of acute kidney injury, bowel injury, acidosis, heart failure and pulmonary edema may occur. In the case of the adult cardiac population, maintenance of ABP above LLA was associated with improved multi-organ outcomes (18), but this cannot be applied to the neonatal case due to differences in neonatal myocardium and peculiarities related to shunted circulation in newborns with congenital heart disease.

Neonates undergoing cardiac surgery are at risk of a distinct low cardiac output state/syndrome (LCOS) about 6–18 h post-operatively. When LCOS is accompanied by high SVR cardiovascular collapse can result. This was noted in the Norwood procedure for hypoplastic left heart syndrome which historically had perioperative morbidity of 50% within the first 48 h of surgery (29). The initiation of afterload reduction via phenoxybenzamine (a vasodilator) during Norwood procedure resulted in lower indexed SVR, higher SVO_2_ via internal jugular oximetric measurements, decreased arteriovenous O_2_ content differences, and improved systemic blood flow (30, 31). Study has shown that freedom from circulatory collapse at 72 h was increased with phenoxybenzamine (95 vs. 69% without phenoxybenzamine (16). The PRIMACORP study in 2003 showed that milrinone (a vasodilator) also decreased the incidence of LCOS after biventricular repair with CPB (32). The need to reduce SVR to prevent LCOS and the need to raise SVR to achieve ABP above the LLA are diametrically opposed clinical goals in the neonates with cardiac disease. Understanding the effect of optimizing ABP based on LLA vs. the rate of LCOS and reduced survival cannot be inferred from adult trials.

### The Challenge of Demonstrating Efficacy of Brain Protection in Neonates

If the burden of hypotension can be effectively reduced by autoregulation monitoring, will the burden of neurologic injury be similarly reduced, and how can that be demonstrated? In the adult RCT of autoregulation monitoring for cardiac surgery, multiple outcomes were evaluated. While MRI differences were not observed, patients with autoregulation monitoring experienced a 45% reduction in post-operative delirium compared to controls. (38 vs. 53%, *p* = 0.04) (17, 18).

The outcomes of adult studies cannot serve as a template for neonatal studies. MRI findings in adults are largely embolic while MRI findings in neonates after cardiopulmonary bypass are primarily watershed white matter injuries (WMI) (3, 4). It is not surprising that autoregulation monitoring did not change the incidence of embolic events, but it is conceivable that maintaining ABP above LLA would reduce ischemic WMI. Therefore, MRI will be important outcome measure for the neonatal population. To date, no intervention has reduced WMI seen on MRI in this population.

Overt neurologic injury is uncommonly seen in neonates, even with substantial MRI injury. Therefore, neurologic exam is likely too insensitive to use as an outcome. Delirium cannot be measured with the same precision in neonatal populations as in adult populations, so it is unlikely that a neonatal study could replicate the adult finding of reduced delirium.

Seizure activity occurs in nearly 10% of neonates after cardiac surgery, is most often clinically silent, and is strongly associated with developmental outcome (33, 34). Seizure activity is an attractive outcome for studying efficacy of autoregulation monitoring because the data are low cost and obtained immediately after surgery. Post-operative EEG should be included in any trial of neonatal autoregulation monitoring during cardiac surgery. With a baseline incidence of 10%, a multi-center trial with large sample size would be required to show a reduction in rate of post-operative seizure.

The gold standard neurologic outcome for neonates with neurologic risk is the developmental exam. Any study that collects data for 5 yr and then requires a 5 yr follow up developmental exam becomes a 10-yr study. The most impactful developmental study of neonates with cardiac disease is the Boston circulatory arrest trial, which followed serial developmental exams into the late teen years (35). Even if a substantial neonatal cohort could be recruited in 5 yr with randomization of autoregulation monitoring, the results of developmental testing at 15 yr would require 20 yr of effort. Extensive work done to date on developing autoregulation monitors for neonatal cardiac surgery is little more than foundation for the efforts of a subsequent generation of clinician-scientists.

## Conclusion and a Look to the Future

Teams that care for neonates with cardiac disease have made substantial progress in achieving survival for complex and delicate surgical disease with very little room for error. The current frontier is the unacceptably high rate of neurologic injury in the survivors of neonatal heart surgery. Given the multifactorial vulnerabilities of the neonatal brain perfused by a malformed circulation, it is not likely that any single intervention will eliminate brain injury. Autoregulation monitoring stands at the front of the line with these interventions because low ABP is needed for survival of neonatal heart surgery, and we do not currently know how low we can lower ABP and still perfuse the brain. The most logical way to answer this question is to measure LLA with autoregulation monitoring.

Dynamic autoregulation monitoring has been developed with adequate validity to reliably delineate optimal perfusion pressure for patients with traumatic brain injury. These concepts have been applied to develop non-invasive methods using reflectance NIRS, and the NIRS-based measures have been validated against gold standard measures of LLA in animal models. What remains is clinical translation specific to the neonatal population, demonstrating feasibility, safety, and efficacy. We have outlined challenges peculiar to neonates with heart disease for each of these clinical research steps.

If feasibility cannot be demonstrated with current methods to measure autoregulation, one can ask if this is inability to modulate blood pressure in the setting of cardiac surgery, or if the autoregulation data is coming to slow to delineate LLA in a practical way. In the latter case, the challenge to making autoregulation monitoring faster has been the fundamental frequency of the autoregulation mechanism which acts as a high pass filter for ABP waves having a period of 30 seconds or longer (36). One promising effort to speed up autoregulation monitoring is the co-trending method put forward by Medtronic, Inc. (37). Another approach is to increase the precision of each measure of autoregulation (thereby decreasing the time to reliable LLA determination). This can be done with inducing low frequency blood pressure waves which can be done with a variety of minimal physiologic perturbations (38).

Safety was not only demonstrated in the adult data, but major morbidity including AKI (acute kidney injury) was lower in patients when ABP was above LLA. It is not clear that the same pattern will hold true in neonates. If SVR crisis, renal or bowel injury, respiratory failure, heart failure, arrhythmia or death are higher in study groups where ABP is kept above LLA, then the specific interventions used to manage ABP will have to be examined. Any method to raise ABP in this population has the potential to cause some combination of these complications. Autoregulation monitoring will only prove beneficial and safe if the intervention can maintain ABP above LLA without causing complication related to low cardiac output.

Once feasibility and safety are demonstrated, sample size required to document efficacy can be calculated. It is anticipated that such a trial will be multi-center, and that the outcomes will be a combination of MRI, seizure activity, and developmental follow-up. The stamina required to complete such a study is non-trivial and would likely span two decades of work. Although this study is the optimal pathway to apply evidence-based medicine to clinical practice, there is very little precedent for successful randomized trials of monitoring technologies. Most standard monitoring was adopted despite a failure of trials to demonstrate outcome benefit (39). Perhaps, given the success of the adult trial of autoregulation monitoring, this pattern will be broken, and proper evidence will be obtained for the rollout of a technology with potential to benefit such a vulnerable population.

## Author Contributions

All authors contributed to the article and approved the submitted version.

## Funding

This research was supported by grant from Additional Ventures: Single Ventricle Research Fund 2020.

## Conflict of Interest

The authors declare that the research was conducted in the absence of any commercial or financial relationships that could be construed as a potential conflict of interest.

## Publisher's Note

All claims expressed in this article are solely those of the authors and do not necessarily represent those of their affiliated organizations, or those of the publisher, the editors and the reviewers. Any product that may be evaluated in this article, or claim that may be made by its manufacturer, is not guaranteed or endorsed by the publisher.
